# Supplementary material for: Estimated 24 h Urinary Sodium-to-Potassium Ratio Is Related to Renal Function Decline: A 6-Year Cohort Study of Japanese Urban Residents
Source: Int J Environ Res Public Health. 2020 Aug 11;17(16):5811. doi: 10.3390/ijerph17165811 (PMC7459630; doi:10.3390/ijerph17165811)
Supplement: Supplementary file 1 [file ijerph-17-05811-s001.zip › ijerph-875944-supplementary 2020.8.11/Figure S1.pdf]

International Journal of
Environmental Research and Public Health

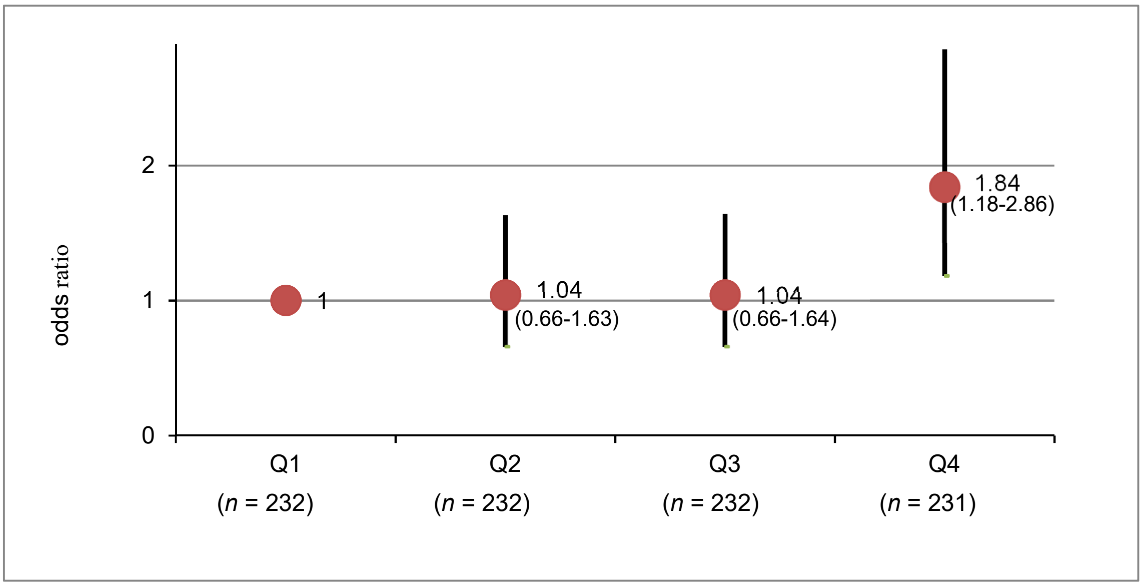

Figure S1. Multivariate-adjusted odds ratio for eGFR decrease according to the quartile groups of UNa/K levels. Q1: UNa/K<1.3; Q2: 1.3 ≤ UNa/K < 1.9; Q3: 1.9 ≤ UNa/K < 2.6; Q4: UNa/K ≥ 2.6. Data are odds ratio and 95% confidence interval. Logistic regression models were used. Adjusted for sex, age, BMI, cigarettesmoking (current/past/none), alcohol drinking (current/past/none), HDL-C, LDL-C, HbA1c, eGFR (baseline), hypertension. eGFR: estimated glomerular filtration rate, BMI: body mass index, HDL-C: high-density lipoprotein cholesterol, LDL-C: low-density lipoprotein cholesterol, hypertension : SBP ≥ 130 or/and DBP ≥ 80.
