# Supplementary material for: Estimated 24 h Urinary Sodium-to-Potassium Ratio Is Related to Renal Function Decline: A 6-Year Cohort Study of Japanese Urban Residents
Source: Int J Environ Res Public Health. 2020 Aug 11;17(16):5811. doi: 10.3390/ijerph17165811 (PMC7459630; doi:10.3390/ijerph17165811)
Supplement: Supplementary file 1 [file ijerph-17-05811-s001.zip › ijerph-875944-supplementary 2020.8.11/Figure S2.pdf]

International Journal of
Environmental Research and Public Health
MDPI

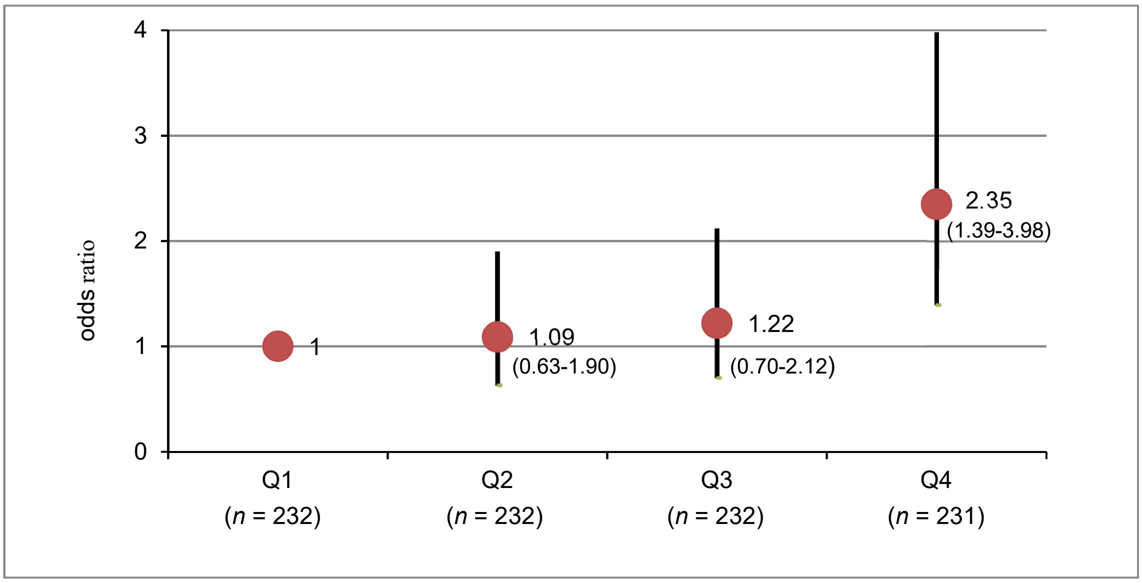

Figure S2. Multivariable-adjusted odds ratio for eGFR decrease according to the quartile groups of e24hUNa/K levels (eGFR decrease was set as 10% decline of eGFR of each participant: mean absolute decline -15.1% in six-year follow-up). Q1: e24hUNa/K < 2.8; Q2: 2.8 ≤ e24hUNa/K < 3.2; Q3: 3.2 ≤ e24hUNa/K < 3.6; Q4: e24hUNa/K ≥ 3.6. Data are odds ratio and 95% confidence interval. Logistic regression models were used. Adjusted for sex, age, BMI, cigarette smoking (current/past/none), alcohol drinking (current/past/none), HDL-C, LDL-C, HbA1c, eGFR (baseline), hypertension. eGFR: estimated glomerular filtration rate, BMI: body mass index, HDL-C: high-density lipoprotein cholesterol. LDL-C: low-density lipoprotein cholesterol, hypertension: SBP ≥ 130 or/and DBP ≥ 80.
