# Supplementary material for: Estimated 24 h Urinary Sodium-to-Potassium Ratio Is Related to Renal Function Decline: A 6-Year Cohort Study of Japanese Urban Residents
Source: Int J Environ Res Public Health. 2020 Aug 11;17(16):5811. doi: 10.3390/ijerph17165811 (PMC7459630; doi:10.3390/ijerph17165811)
Supplement: Supplementary file 1 [file ijerph-17-05811-s001.zip › ijerph-875944-supplementary 2020.8.11/Table S1.pdf]

International Journal of
Environmental Research and Public Health

MDPI

Table S1. Multivariate-adjusted means of eGFR and its 6-year change according to the quartile groups of UNa/K levels.

	Q1 (*n* = 232)		Q2 (*n* = 232)		Q3 (*n* = 232)		Q4 (*n* = 231)		*p*-Value
	Mean	(95% CI)	Mean	(95% CI)	Mean	(95% CI)	Mean	(95% CI)	
Egfr (6 years later), mL/min/1.73m²	75.0	(74.5 – 75.5)	74.6	(74.1 – 75.1)	74.5	(74.0 – 75.0)	73.4	(72.9 – 73.9)	<0.001
eGFR amount of change, mL/min/1.73m²/year	-0.71	(-0.80 – -0.62)	-0.77	(-0.86 – -0.69)	-0.80	(-0.88 – -0.71)	-0.97	(-1.06 – -0.88)	<0.001
change rate in eGFR, %/year	-0.88	(-1.01 – -0.76)	-0.97	(-1.09 – -0.85)	-1.01	(-1.13 – -0.90)	-1.24	(-1.36 – -1.12)	0.001

Q1: UNa/K < 1.3; Q2: 1.3 ≤ UNa/K<1.9; Q3: 1.9 ≤ UNa/K < 2.6; Q4: UNa/K ≥ 2.6; Data are presented as mean and 95% confidence interval. Analysis of variance were used to adjust for sex, age, BMI, cigarettes smoking(current/past/none), alcohol drinking (current/past/none), HDL-C, LDL-C, HbA1c, eGFR (baseline), hypertension. *p*-Values for difference between groups. eGFR: estimated glomerular filtration rate, BMI: body mass index, HDL-C: high-density lipoprotein cholesterol, LDL-C: low-density lipoprotein cholesterol, hypertension: SBP ≥ 130 or/and DBP ≥ 80.
